# Late enhancement findings in a prospective study concerning late functional outcomes following a Ross Procedure

**DOI:** 10.1186/1532-429X-11-S1-P98

**Published:** 2009-01-28

**Authors:** Rod Jones, Raj Puranik, Wendy Norman, Victor Tsang, Vivek Muthurangu, Johannes Nordmeyer, Philip Lurz, Graham Derrick, Fiona Walker, Shay Cullen, Philip Bonhoeffer, Andrew M Taylor

**Affiliations:** 1 UCL Institute of Child Health, London, UK; 2 Great Ormond Street Hospital, London, UK; 3 The Heart Hospital, UCLH, London, UK

**Keywords:** Aortic Valve Disease, Late Enhancement, Ross Procedure, Scar Burden, Ross Operation

## Background

In patients with aortic valve disease, long-term autograft and/or homograft durability post Ross procedure, remains controversial. Recent studies indicate a late re-intervention rate of up to 40%, compared to that of less than 25% published in earlier studies.

The long-term prospective data regarding functional outcomes in this patient group is also limited. We also aim to describe the extent of scar burden within the myocardium using late gadolinium MR imaging after the Ross procedure.

## Methods

60 subjects (22.9 y, range 6.4–52.2 y) who are in active clinical follow-up and underwent the Ross procedure between 1994 and 2006 (8.1 y post Ross operation, range 1.8–14.0 y) were prospectively assessed.

A multi-modality approach was employed, with subjects performimg cardiopulmonary exercise testing, 2D-echocardiography and cardiac MR imaging (including late enhancement).

Late enhancement scar imaging within the myocardium was performed with segmented phase-sensitive inversion recovery sequences (Image parameters – TR = 2 × RR interval; TE = 3.4 ms; flip angle = 25°; slice thickness = 10 mm; matrix = 144 × 256; field of view = 300–380 mm, acquired during a single breath-hold) 10 minutes post administration of intravenous contrast (0.1 mmol/kg of gadolinium pentatate, Magnevist). Imaging included the entire short-axis and long axis planes. A 'Look-locker' sequence was used to determine the inversion time, which reflected the null point of the normal myocardium. The volume of scar within the myocardium was expressed as a % of the total LV volume.

## Results

**Figure 1:**
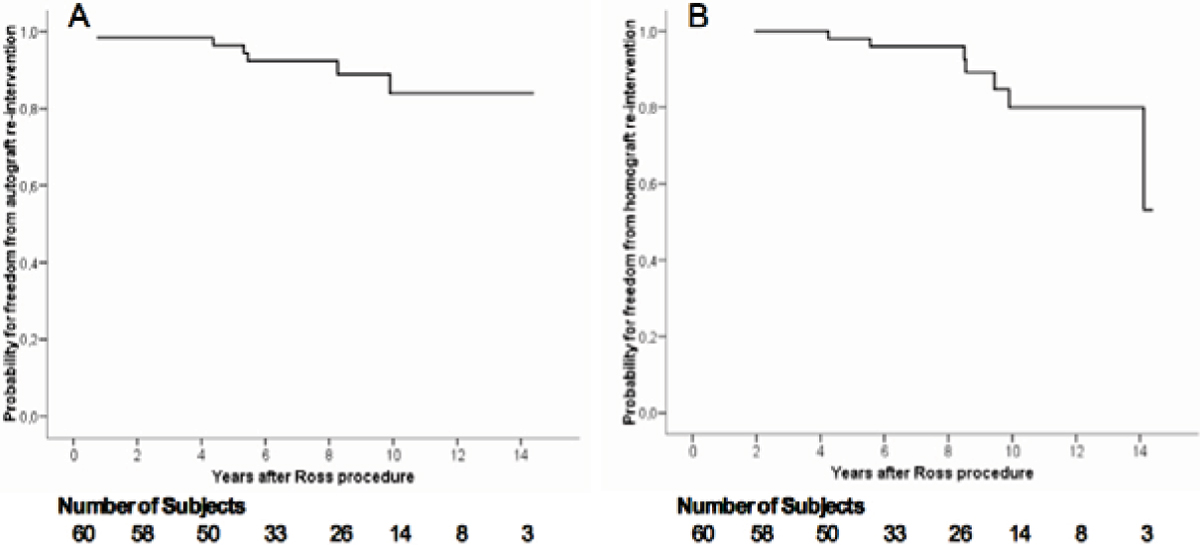
**Probability of freedom of re-intervention at 10 years after the Ross Procedure**. Data presented as Kaplan Meier plots demonstrating the probability of freedom from re-intervention for the autograft (Panel A) and the homograft (Panel B). The total number of subjects relative to the years after the Ross procedure is shown.

80% of subjects had aortic stenosis as their original diagnosis (43% biscuspid aortic valves). The probability of freedom from re-intervention on the autograft and the homograft at 10 years was 84% and 80% respectively (Figure 1). On exercise testing, the mean exercise capacity achieved was 87 ± 23% of predicted. On 2D-echocardiography the peak velocity across the autograft and homograft was 1.3 ± 0.4 m/sec and 2.6 ± 0.6 m/sec respectively. Cardiac MR imaging identified only trivial mean autograft and homograft regurgitation (5.9 ± 7.6% and 6.4 ± 9.3% respectively). Biventricular systolic function was normal (LV EF 63 ± 6% and RV EF 61 ± 7%).

55/60 subjects underwent delayed enhancement studies with intravenous gadolinium. 18/55 subjects (33%) had evidence of myocardial scar within the LV (Table 1). The mean scar volume within the LV was 6.0 ± 5.0 cm3 and mean % of scar within the left ventricle was 5.4 ± 8.5%. In 5/9 cases of septal wall scar the surgeon had commented on bleeding at the time of autograft harvest. There was no significant relationship between LV scar and baseline ECG abnormalities (p = 1.0).

**Table 1:** Table 1

Pts	Gender	Age	Years post Ross	Scar Vol (cm^3^)	Scan as % of LV volume	Pattern within LV	Surgical aspects
1	F	48.4	5.2	1.9	1.8	Apex (SE)	N/A
2	M	52.1	8.8	9.1	9.9	Mid to distal septum (SE), Apex (SE)	N/A
3	M	19.3	5.8	3.3	1.9	Basal septum (FT)	Bleeding at autograft harvest
4	F	22.3	5.7	3.1	3.0	Basal septum (SE)	N/A
5	M	16.7	8.2	1.9	1.4	Apex (FT)	N/A
6	M	20.7	5.6	7.8	4.8	Basal septum (FT)	Bleeding at autograft harvest
7	F	33.8	5.5	6.3	4.5	Basal septum (FT) and Apex (FT)	N/A
8	F	32.8	8.7	2.9	1.4	Apex (FT)	N/A
9	M	34.6	6.2	0.9	0.5	Mid septum (SE)	Anomolous coronary anatomy, no bleeding
10	M	6.4	5.8	17.3	37.6	Complete ring enhancement of LV SE	Endocardial fibroelastosis
11	M	22.3	6.2	2.5	1.9	Basal lateral wall (FT)	N/A
12	M	24.7	10.3	19.0	10.2	Antero-septal (FT)	Small branch coronary vessel divided at autograft harvest
13	M	26.7	12.2	6.9	3.9	Basal septum (FT) and Apex (SE)	Bleeding at autograft harvest
14	M	27.1	13.4	1.9	1.1	Apex (SE)	Minor bleeding at autograft harvest
15	M	25.2	13.0	4.0	2.8	Apex (SE)	N/A
16	M	23.3	12.0	7.2	4.4	Antero-septal (FT)	Small branch coronary vessel divided at autograft harvest
17	M	38.5	13.9	4.9	3.1	Inferior-posterior (SE)	N/A
18	M	24.7	13.9	6.7	3.5	Inferior (SE)	N/A

SE = subendocardium, FT = full thickness.

## Conclusion

Results show, many years after the Ross procedure, there is excellent autograft and homograft function, translating into high functional capacity. Institution based re-intervention rates of less than 20% are in agreement with earlier published work.

We provide the first description of the extent of scar burden within the LV myocardium using late gadolinium MR imaging after the Ross procedure. The predominant distribution of LV scar was identified within the LV septum (50% of cases). This is likely mainly related to technical aspects surrounding the autograft harvest, site's proximity to septal coronary vessels.

Although in our study we found no association between LV scar burden and ECG abnormalities, the prognostic significance of these findings may only be truly deciphered after longer-term follow-up.

